# Patient Voices: What Can We Learn From the Covid‐19 Pandemic About Follow‐Up Care in Gynaecologic Oncology?

**DOI:** 10.1111/hex.70405

**Published:** 2025-09-22

**Authors:** Esther M. Vermaas, Luc R. C. W. Van Lonkhuijzen, Henrike Westerveld, Maaike A. Van Der Aa, Tijmen J. Bonestroo, Brigitte F. M. Slangen, Johanna W. M. Aarts

**Affiliations:** ^1^ Department of Obstetrics and Gynaecology, Division of Gynaecologic Oncology Amsterdam UMC location Vrije Universiteit Amsterdam Amsterdam the Netherlands; ^2^ Cancer Centre Amsterdam, Treatment and Quality of Life Amsterdam the Netherlands; ^3^ Department Research & Development The Netherlands Comprehensive Cancer Organization Utrecht the Netherlands; ^4^ Department of Radiotherapy, Erasmus Medical Cancer Institute University Medical Centre Rotterdam the Netherlands; ^5^ Department of Obstetrics and Gynaecology Rijnstate Hospital Arnhem the Netherlands; ^6^ Department of Obstetrics and Gynaecology Maastricht University Medical Centre (MUMC+) Maastricht the Netherlands; ^7^ GROW‐ School for Oncology and Reproduction Maastricht the Netherlands

**Keywords:** aftercare, cancer survivorship, cervical cancer, Covid‐19, endometrial cancer, follow‐up, gynaecologic oncology, ovarian cancer, patient perspective, vulvar cancer

## Abstract

**Purpose:**

To explore women's experiences with follow‐up care after gynaecological cancer during the Covid‐19 pandemic and identify key elements of aftercare from their perspective.

**Methods:**

A qualitative study was performed, including five focus group discussions and two individual interviews with 20 participants diagnosed with ovarian (*n* = 5), cervical (*n* = 6), endometrial (*n* = 5) or vulvar cancer (*n* = 4) who received follow‐up care during the Covid‐19 pandemic in the Netherlands. Transcripts underwent thematic analysis, guided by the framework of the Picker Principles of Patient‐Centred Care.

**Results:**

Five themes were generated: (1) continuity of care, (2) absence of family members and carers, (3) meeting my needs, (4) managing my needs and (5) the cancer survivor narrative. The main changes experienced during the Covid‐19 pandemic were the introduction to remote healthcare and the absence of family members. An interconnection between themes was found, highlighting that providing a designated, always accessible contact person can be a catalyst for the improvement of information provision and healthcare guidance.

**Conclusions:**

In conclusion, this study highlights the need for personalised and patient‐centred follow‐up that promotes patient empowerment, and how this can be provided by a designated contact person. Findings emphasise the importance of tailored support, involvement of family members, addressing information gaps, and overcoming barriers to self‐management. Lastly, the findings provide direction on how to approach follow‐up care in the future.

**Patient Contribution:**

This study was done in close collaboration with the patient advocacy group Olijf; their significant input in both the design and conduct of the study is invaluable. Olijf's involvement ensured that the research remained patient‐centred and aligned with the real‐life concerns and priorities of those affected by gynaecological cancers. The participants in this study, all of whom are gynaecological cancer survivors, played an important role by sharing their experiences, and we extend our gratitude to them. Their insights were critical in shaping the findings of this research.

## 1 Introduction

In the Netherlands, around 5000 women^1^ are diagnosed with gynaecological cancer annually, and that number is increasing [1, 2]. Once patients have completed cancer treatment, they are monitored for a period of time, which is referred to as follow‐up [3, 4]. The proposed objectives of follow‐up are early detection of possible disease recurrences and the provision of supportive care [4, 5].

Traditionally, follow‐up is planned in a predetermined schedule of hospital visits. These consultations with a medical specialist include an assessment of signs and symptoms, physical examinations, and, in some cases, additional medical examinations. In accordance with international guidelines, this schedule generally includes visits every 3 months in the first and second year after primary treatment, every 6 months in the third and fourth year, and annually thereafter [6, 7, 8, 9, 10]. This schedule is similar for all types of gynaecological cancer and usually completed 5 years after treatment, although patients with vulvar cancer might require lifelong follow‐up [6, 7, 8, 9, 10]. This form of follow‐up is often coined as routine hospital‐based follow‐up (HFU). However, it is unclear if HFU contributes to improved survival, quality of life and whether it is cost‐effective [3, 4, 5, 11, 12, 13, 14, 15, 16, 17, 18]. Moreover, studies in the Netherlands and internationally have shown that follow‐up care for women with gynaecological cancer often fails to meet their emotional, informational and supportive care needs, impacting their overall quality of life [19, 20, 21, 22, 23, 24].

The increasing scarcity of clinical resources may limit the feasibility of this labour and resource‐intensive approach in the future [25]. There may be alternatives to HFU that offer similar or better results. These include reducing the frequency of hospital visits [26, 27], involving nurses [28, 29, 30, 31], incorporating telemedicine [32, 33, 34], and patient‐initiated follow‐up (PIFU) [35]. High‐quality care should not only be safe and effective, but also provide patient‐centred care (PCC). Therefore, it is important to involve patients when designing new follow‐up strategies [36].

During the Covid‐19 pandemic, many conventional medical practices were upended to reduce the transmission of the virus. In gynaecologic oncology, studies have documented a reduction in in‐person follow‐up visits, an increased reliance on remote consultations, and decreased availability of healthcare personnel [34, 37, 38, 39, 40]. These shifts prompted the introduction of innovative models of care delivery. This unique period provides an opportunity to evaluate the experiences of patients with novel forms of follow‐up care and to explore patient preferences and needs.

The aim of this qualitative study was to explore how women experienced follow‐up care for gynaecological cancer during the Covid‐19 pandemic. In addition, the overall patient's perspective on general aspects of aftercare was explored to identify key elements of what they consider important.

## 2 Methodology

### 2.1 Study Design

This study used a qualitative descriptive design. Data were collected through focus group (FG) discussions to explore patients' perspectives and experiences. This method provides interactive and dynamic benefits, for it allows participants to express their own perspectives and experiences whilst engaging in a collective dialogue that facilitates mutual understanding and deeper reflection on the topics [41]. Ethical approval was granted by the Ethics Committee of the Amsterdam University Medical Centre (#FWA00032965). The COnsolidated criteria for REporting Qualitative research (COREQ) checklist was used for this report (Supporting Information 1) [42].

### 2.2 Setting and Participants

Patients were eligible for participation if they had been treated for ovarian, cervical, endometrial or vulvar cancer and subsequently followed up for at least 1 year during the Covid‐19 pandemic (between March 2020 and March 2022). Participants needed to have full comprehension of the Dutch language and be willing to participate in online FGs. Participants were recruited via three pathways: (1) public calls through the Dutch gynaecological cancer patients' association, Olijf, who posted an appeal on their website and newsletter, (2) flyers placed in the waiting rooms of several gynaecologic oncology centres, and (3) direct invitation by their treating clinicians. Interested individuals could contact the research team directly via email or fill out an online form, or clinicians could relay their contact details with consent. Those who expressed interest were contacted to confirm eligibility and were provided with a recruitment letter containing information about the study and an informed consent form. In the week before the FG, reminder e‐mails were sent, containing a hyperlink to access the online platform and instructions on how to log in.

### 2.3 Data Generation

Three experienced and impartial moderators alternated in pairs to moderate FGs. Discussions were guided by a topic list (Supporting Information 2). The participants were grouped based on their tumour type; we aimed to organise at least one FG per tumour type. In total, five FGs were held. The first four were organised by tumour type. We conducted a fifth FG open to participants with all eligible tumour types. This final group included newly recruited participants and served to ensure data consolidation and confirm data saturation was reached. Each FG lasted between 60 and 90 min. Two individual interviews were conducted with participants who were unable to participate in their originally assigned FG. All FGs and interviews were audio‐recorded. Recordings were transcribed verbatim by one member of the research team (X.B.) and reviewed by another (E.V.). Transcripts were not returned to the participants to ensure privacy. Data generation and analysis were conducted concurrently, allowing for emergent concepts and themes to be incorporated in the study.

### 2.4 Data Analysis

This study used the six‐step thematic analysis procedure as proposed by Braun and Clarke: (1) familiarising with the data, (2) systematic data coding, (3) generating initial themes, (4) developing and reviewing themes, (5) refining, defining and naming themes, and (6) writing the report [43]. We used an inductive approach to explore and conceptualise the data. To guide data analysis and interpretation, the framework of the eight Picker Principles PCC was used in the interpretive phase, providing a lens to add depth and meaning to the data. The eight Picker Principles are: (1) continuity of care and smooth transitions, (2) involvement and support for family and carers, (3) effective treatment delivered by trusted professionals, (4) involvement in decisions and respect for preferences, (5) fast access to reliable healthcare advice, (6) clear information, communication and support for self‐care, (7) emotional support, empathy and respect, and (8) attention to physical and environmental needs [44]. Two researchers (E.V. and X.B.) conducted an open coding process using MaxQDA [45]. Transcripts were independently explored and coded, and related codes were grouped into themes. Subsequently, the researchers jointly reviewed the coded transcripts to identify areas of convergence and divergence. To finalise the generated themes, a senior researcher (J.A.) conducted a review of the thematic analysis. Participants did not provide feedback on these findings.

## 3 Results

### 3.1 Participant Characteristics

A total of 22 patients were assigned to participate in a FG. Two patients declined participation after the FG assignment due to personal health‐related circumstances. In total, 20 patients participated in this study. Participants were treated for ovarian (*n* = 5), cervical (*n* = 6), endometrial (*n* = 5) or vulvar cancer (*n* = 4). Participants underwent treatment and subsequent follow‐up care in eight different hospitals from various regions of the Netherlands. All patients received part of their follow‐up care during the Covid‐19 pandemic, for at least a year.

### 3.2 Overview of Generated Themes

**Figure 1:**
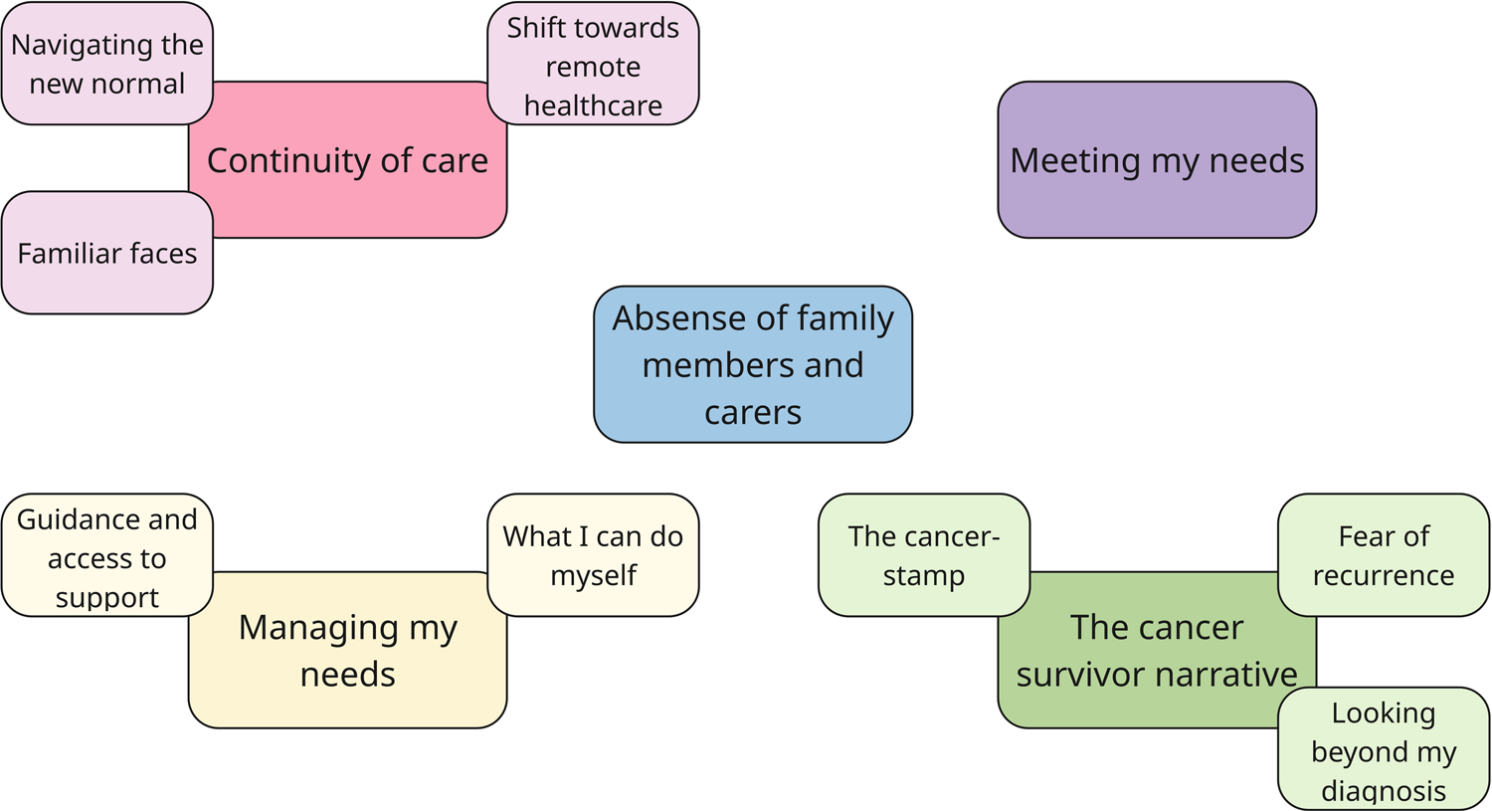
Overview of themes. This figure presents the five themes with corresponding sub‐themes.

Five themes were generated from data analysis: (1) continuity of care, (2) absence of family members and carers, (3) meeting my needs, (4) managing my needs and (5) the cancer survivor narrative. These are presented in Figure 1.

#### 3.2.1 Theme 1: Continuity of Care

##### 3.2.1.1 Navigating the New Normal

Participants generally expressed understanding of the evolving Covid‐19 situation and the challenges the healthcare system faced in adapting. Most accepted changes, such as the inability to choose between remote or in‐person consultations, as well as changes to the frequency of their follow‐up appointments. Many reported that their consultation schedules were established early and followed with minimal disruptions. However, some appeared to contradict themselves, initially stating they did not experience a reduction in follow‐up consultations, but stating later on that they did have fewer consultations.

So that [first follow‐up consult when the COVID‐19 pandemic started] did not go ahead in‐person because of corona, but became a telephone appointment.

P15

##### 3.2.1.2 Shift Towards Remote Healthcare

A widespread shift to remote consultations using telephone calls was noted, though the extent varied. While some switched predominantly to remote follow‐up, others experienced only occasional telephone consultations, often to discuss test results or related matters. Responses to telephone consultations were mixed. Satisfaction with telephone consultations depended on specific factors, including adherence to appointment times to allow for the presence of family members, sufficient time for consultations, and attentive care. Though initially met with discomfort, remote consultations became more acceptable over time, particularly as patients gained confidence in recognising symptoms that would require in‐person attention. Several described a process of gradual adaptation:

[a telephone consultation] doesn't matter that much, […] I think I now have sufficient tools and sufficient certainty to be okay with it.

P15

[Telephone consultations] also takes some getting used to […], once you get into the rhythm it's not such a disaster.

P13

##### 3.2.1.3 Familiar Faces

Continuity of personnel emerged as a key factor in maintaining trust and comfort. Even with protective face masks obscuring faces during the Covid‐19 pandemic, participants preferred consultations with familiar providers. Seeing the same doctor or nurse contributed to a sense of stability and personalised care. Some reported the presence of the same nurse specialist during consultations, to stay up‐to‐date about the patient. This nurse also served as their contact person, ensuring a seamless flow of information between appointments and acting as a resource for addressing problems or questions. Conversely, participants involved with multiple healthcare providers often expressed dissatisfaction and confusion. Uncertainty about who to contact and inconsistent information across providers were common concerns, indicating a lack of alignment between providers.

And I find that boundary [between different healthcare professionals]: [who] to go with which complaints. That has not always been clear to me.

P9

#### 3.2.2 Theme 2: Absence of Family Members and Carers

During the several pandemic peaks, participants were not allowed to bring their family members or other carers to follow‐up appointments. While some participants complied with these rules, others defied hospital rules and brought someone along, often without objections from hospital staff. For many, their follow‐up experiences included both types of appointments—those attended alone and those with a support person present. Having experienced both scenarios, participants were able to clearly articulate the value of being accompanied. They described benefits, such as emotional support, increased participation during consultations, help with asking questions, better information retention, and relief from having to recap the appointment afterwards. Attending alone was frequently described as emotionally taxing. Participants expressed heightened feelings of vulnerability and distress:

So, if there's another pandemic, and you ask, “What is good follow‐up?” I think it would be really nice if your partner is allowed to come. It did not make me any worse, but I did find it terribly nerve‐wracking to go through it all alone.

P13

#### 3.2.3 Theme 3: Meeting My Needs

Participants' experiences were related to the extent of personalisation of their follow‐up care and the extent to which they were involved in decisions about it. While many described their care as tailored to their needs, others found it generic, structured around a standardised care pathway with little room for individualisation. Some participants reported that their follow‐up followed a fixed schedule, seemingly applied uniformly to all patients, without accounting for individual preferences or needs:

But isn't that also the most difficult thing about follow‐up? […] follow‐up was fairly the same for everyone, it is a fixed process. But every person needs different help.

P3

In contrast, others described being actively involved in decisions about their care. They reported collaborative interactions with healthcare professionals that allowed for shared decision‐making regarding various aspects of their care, including the frequency of appointments, the mode of consultations and the choice of healthcare provider:

It is very nice […] that you can indicate yourself: is it possible to do [consultations every] 4 months now? Or do you still want three? That you have the feeling that you have a say in this. And then you may send us down a different path, but it is important that we at least have the feeling that we are in control of it.

P1

#### 3.2.4 Theme 4: Managing My Needs

##### 3.2.4.1 Guidance and Access to Support

Although participants generally praised their treatment experience, most expressed concerns after initial treatment. They reported feeling abandoned and left to navigate their situations on their own:

…[during cancer treatment] there was attention for a psychologist, for a social worker, a doctor that listened to you, partner was asked how they were doing.… But then, once you're done, it's gone and you're kind of left to your own devices.

P8

Several experienced they needed to rely on themselves to manage the care they needed that was delivered outside the hospital, including physiotherapy and mental health support. Some participants noted a lack of guidance from healthcare providers in organising the care they needed. They emphasised the need for assistance and proactive support, pointing out that no one was actively asking about and addressing their specific needs. This was heightened during the Covid‐19 pandemic, although participants also reported experiencing this pre‐ and post‐pandemic. Some felt they only received needed care when they took a proactive stance in requesting it, believing they would not receive care if they did not advocate for themselves:

To who do you turn and who do you ask [questions]? And if you are not assertive yourself, nothing will happen. There is no one who calls you and says: do you need help or have you already thought about this, or do you need this or that. You have to indicate it all yourself and if you don't, nothing will happen.

P8

This lack of guidance extended to information provision. While some felt well‐informed about follow‐up and the specifics associated with their disease, others felt that the information was presented in an understandable manner. Others reported feeling inadequately informed about what to expect after treatment. They lacked knowledge about where to seek information concerning health‐related issues or important signs and symptoms of recurrence to watch for. Several participants highlighted the challenge of finding reliable sources or platforms to gain information about post‐treatment care.

I had to ask about everything. Little information is given if you don't specifically ask.

P13

The presence or absence of a clear point of contact made a major difference for participants. An important factor was the availability of an easy communication channel with the hospital, to enable quick access to assistance when needed. Many appreciated having a designated contact person, often a nurse specialist, who could facilitate access to information, connect them with their gynaecologist, or provide referrals tailored to their needs:

[It] was always made clear to me. If there is anything, you can always contact us, so yes, I felt quite safe.

P19

In contrast, some lacked a clear contact person and were unsure whom to turn to. This led to frustration, hesitance to reach out, and unmet needs:

I also missed a point of contact. I didn't want to disturb the doctor actually. And yes, then you could call the [hospital department's] desk, but then I didn't always have very knowledgeable people to talk to. So at some point I didn't do that [anymore].

P7

##### 3.2.4.2 What I Can Do Myself

Some participants expressed that their follow‐up focused on self‐care strategies to minimise potential complaints. Healthcare providers educated them on which specific symptoms to monitor at home. Participants found this aspect of self‐care empowering and reassuring:

‘[Follow‐up care] is also very much focused on: what can you do yourself, […] what can you do yourself to reduce certain symptoms.

P12

Some with vulvar cancers were taught by their healthcare provider how to perform physical examinations at home for self‐evaluation.

My doctor, he just said: this is a type of cancer that tends to come back, so he also again paid a lot of attention to also keep checking yourself.

P16

#### 3.2.5 Theme 5: The Cancer Survivor Narrative

##### 3.2.5.1 The Cancer‐Stamp

Throughout the discussions, participants demonstrated a shared understanding of what it means to be a cancer survivor. One participant introduced the concept of having the ‘cancer‐stamp’ as a metaphor to symbolise societal perceptions on the lived experiences of cancer survivors that influence how others interact with her. For instance, during the pandemic, she explained that her ‘cancer‐stamp’ could open doors, as healthcare professionals were more lenient when enforcing restrictions on her.

[…] well I always call it the “cancer‐stamp,” it [sounds] very unpleasant maybe. But I had the “cancer‐stamp” and I was allowed everything in the hospital. I was allowed to come with my partner, no weird questions were asked, I was just allowed to go in there as a couple.

P4

##### 3.2.5.2 Fear of Recurrence and Seeking Reassurance

Fear of recurrence was a pervasive theme, often linked to the broader issue of not trusting their body after a cancer diagnosis. For many, physical symptoms, however minor, could activate a deeply ingrained anxiety:

That little voice in the background is still always there.… so much has happened in that belly with [cancer treatment], you just always have pains, and complaints, and rumblings. Because you know 1 day is not the other. And with us [cancer survivors] that little antenna is immediately extended: surely it won't be [cancer recurrence]

P3

However, some were able to put this in perspective, noting that over time their fears diminished and they gained a sense of security and confidence. The majority of our participants noticed that their fear and anxiety were often connected to an upcoming follow‐up appointment:

And even though you know, I had no complaints at all, and yet [a follow‐up appointment] is very nerve‐wracking every time.

P13

Participants placed a high value on in‐person follow‐up appointments with physical examinations and diagnostic tests, considering them as confirmation that their disease had not relapsed:

I am also very much of the certainty, of the knowledge, of every time those markers and the internal [physical] examination. I need that and got that every time.

P5

The absence of such examinations left participants feeling insecure. Despite concerns about Covid‐19 infections, participants prioritised attending appointments in person to ensure that these examinations could take place.

##### 3.2.5.3 Looking Beyond My Diagnosis

Participants emphasised the importance of effective communication and connection with their healthcare providers. Participants valued providers who initiated and encouraged discussions on topics important to them personally, beyond cancer‐related concerns. This holistic approach, which demonstrated a genuine interest in the patients' well‐being and home situations, left participants feeling reassured and confident in their provider.

She [the nurse] completely put my mind at ease. And she really took time for the conversation and included many more things. In the sense of how [my family] responded at home, what I could and couldn't do, whether I was cared for at home, what kind of agreements I had with my work, about the pain relief. She made it so broad that I really thought: wow, what is this? Very nice indeed. At the time I didn't have that question, but afterwards I thought: oh, this is very good. I actually really liked that.

P12

#### 3.2.6 Interconnections Between Themes

**Figure 2:**
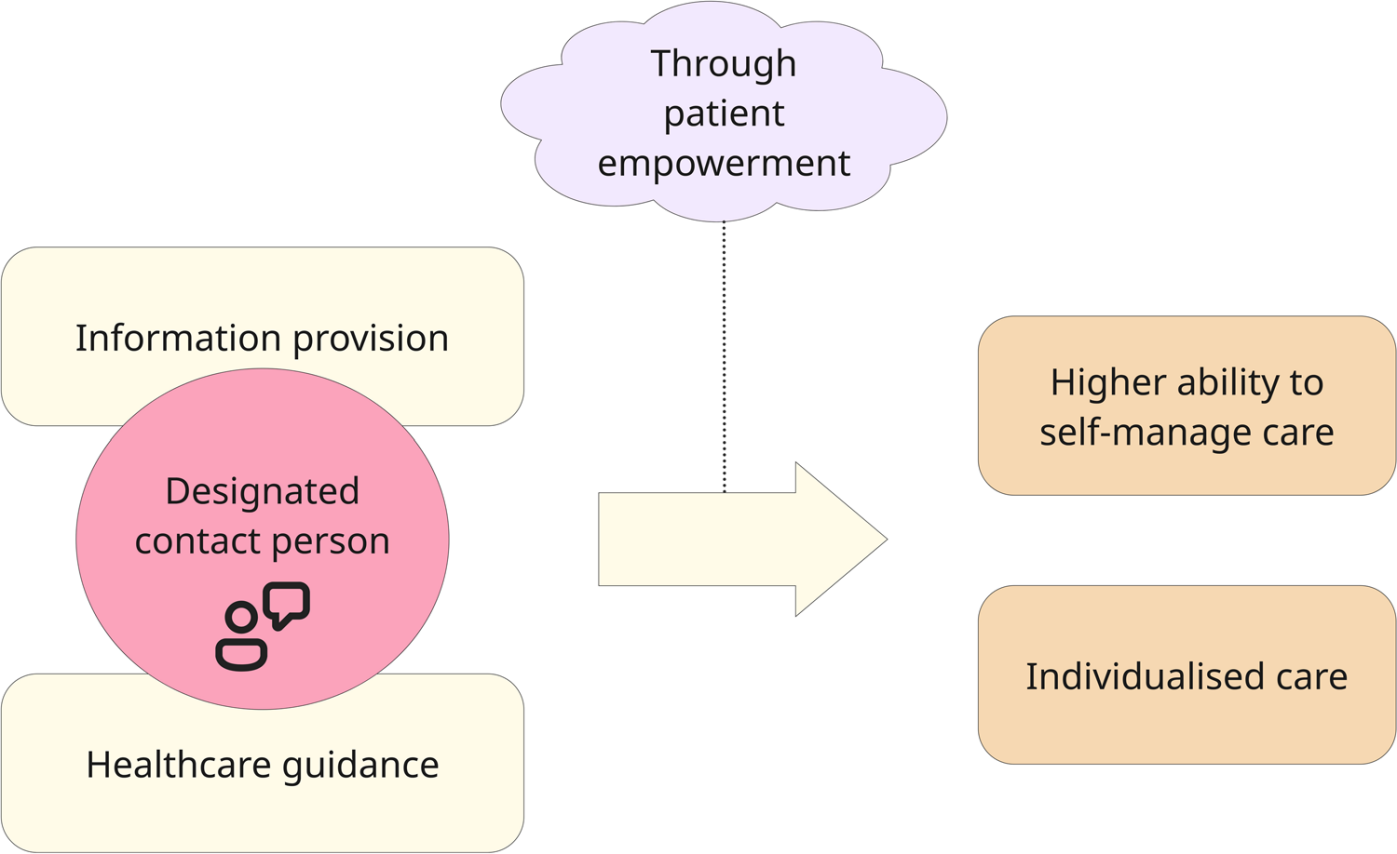
Interactions between themes. A person of contact can be a catalyst, providing information and healthcare guidance, improving the patient's ability to self‐manage care and providing more individualised care through patient empowerment.

In this study, we identified interactions between positive and negative patient experiences related to various themes. We observed a pattern among participants who reported feeling more isolated and dissatisfied, extending to challenges in independently organising care. These participants also reported a lack of a designated contact person, feeling the need to rely on themselves to manage their healthcare needs, and not receiving adequate guidance. In contrast, participants who reported having a designated contact person who was always accessible to them, such as a case manager or nurse specialist, had more positive experiences. They reported receiving satisfactory healthcare guidance and information and expressed having the ability to self‐manage their care. In summary, we found that providing a designated, always‐accessible contact person can be a catalyst for improving information provision and healthcare guidance, leading to a higher patient ability to self‐manage their care and more individualised care through patient empowerment. Figure 2 visualises the interconnections between these themes.

## 4 Discussion

### 4.1 Main Findings

This study explored the experiences of gynaecological cancer survivors with follow‐up care during the Covid‐19 pandemic and their overall perspectives on aftercare, identifying fundamental patient needs post‐treatment. The challenges faced during the pandemic put emphasis on several aspects of PCC that are important when providing follow‐up care, making the findings of this study particularly insightful. This study illustrates the importance of availability and accessibility in follow‐up care. Follow‐up care should be organised to be more personalised, ensuring it meets individual needs. Those in follow‐up should have access to a designated contact person who can be readily reached. By providing patients with appropriate information resources and education, self‐management can be enhanced. This approach enables the provision of on‐demand care, increases patient empowerment and facilitates more individualised care. Our findings offer insight into how to approach and prioritise care in times of scarcity or major disruption, such as pandemics, highlighting the importance of strategies that ensure care continuity. These should safeguard the involvement of family members and carers and equip patients with tools for effective self‐management.

### 4.2 Strengths and Limitations

The qualitative design allowed an exploration of patients' experiences, revealing both shared and distinctive aspects in survivorship journeys. The study's strength lies in adopting Picker Principles as a guiding framework, enriching the depth of our findings and commitment to patient‐centred research [44]. Limitations include potential for self‐selection bias from recruiting through a patient association, which tends to attract more assertive patients with stronger opinions. Another potential limitation is recall bias, as participants were asked about experiences from 2 years ago. However, this is less applicable for general questions about follow‐up that are not specific to the pandemic. Additionally, most participants were from the same region, which may result in a lack of representation. Two participants were interviewed individually due to their inability to participate online due to a lack of computer skills.

Qualitative research is often subject to criticism based on a limited sample size. Despite reaching data saturation, the same could be argued for this study. Following Braun and Clarke's reflexive thematic analysis, we question the feasibility of achieving data saturation when there are endless possibilities for new insights. We challenge the linkage of ‘meaningfulness’ to specific numbers of data collections, such as sample size. Our main focus was that the data sufficiently addressed our research questions and portrayed a rich, complex and multifaceted story [46].

### 4.3 Interpretation

Our study provides a unique contribution to the existing literature regarding the patients' perspective on follow‐up care. During the pandemic, progress has been made towards remote care. Despite overall satisfaction with telephone consultations, the majority in this study indicated a preference for in‐person consultations. This was influenced by the importance participants attributed to additional examinations, perceiving them as crucial for ruling out recurrence, which is in line with previous studies [47, 48, 49]. This may pose a challenge when transitioning towards remote follow‐up care, such as telemedicine. Successful implementation of telemedicine requires education and active participation of both patients and healthcare providers. Therefore, it is vital to educate patients about the limited role of physical and additional examinations in detecting recurrences and their limited impact on survival [13, 16, 33, 50, 51, 52]. Previous studies support the notion that, despite the high appreciation for examinations, patients expressed a need to enhance self‐management and coping strategies during the survivorship phase [21, 53, 54]. Moreover, telemedicine can be essential in improving self‐management [32, 33, 34]. Interventional studies exploring nurse‐led telephone follow‐up reported similar quality of life and patient satisfaction between groups receiving telephone follow‐up or in‐person HFU [28, 29, 55, 56, 57]. Furthermore, Beaver et al. found that patients tended to prefer what was familiar to them [56]. Therefore, addressing patient perceptions, providing education and considering patient preferences are essential when incorporating telemedicine in follow‐up care.

Our findings shed light on the importance of meeting and managing patient needs, raising concerns about the impact of standardised approaches, and recognising the need for personalised strategies to follow‐up care, as a one‐size‐fits‐all approach may not be universally beneficial and could even lead to negative outcomes [3, 4, 5, 11, 12, 13, 14, 15, 16, 17, 18]. PCC focuses on the patient as an individual, recognising their personal values and unique needs. Furthermore, it focuses on helping patients make informed decisions about their own care. It has been shown to improve patient satisfaction and health outcomes, as well as the effectiveness of the healthcare system [44, 58, 59]. It is important to recognise that challenges in meeting and managing patient needs may become more apparent during periods of healthcare scarcity, such as the Covid‐19 pandemic. Providing PCC can be complex, and multiple facets may interplay, which should be considered to provide meaningful improvements. De Rooij et al. challenged the idea that improving follow‐up care is not solely based on one facet, such as information provision, as they raised the question: ‘Does information heal or hurt?’ They examined the use of Survivorship Care Plans (SCPs)—structured documents intended to improve communication and self‐management by summarising diagnosis, treatment and follow‐up information and recommendations. Contrary to expectations, their study showed that SCPs do not enhance satisfaction with information and care and might even have a negative impact on endometrial and ovarian cancer patients [60]. In our study, we have found elements of follow‐up care that interact, as well as how availability and accessibility influence information provision, healthcare guidance and, in turn, patient self‐management. Integrating SCPs within a broader framework that addresses these aspects may yield more favourable outcomes. Therefore, we propose that these interactions should be considered when providing alternatives to HFU, to improve patient outcomes and enhance the cancer survivorship experience.

Acknowledging the invaluable contributions of nurses, the study advocates for the pivotal role they play, providing a contact person, facilitating referrals through their extensive knowledge of other active healthcare professionals in their region and ensuring care continuity through their consistent presence and involvement. Furthermore, by incorporating non‐disease‐related inquiries into patients' well‐being, their holistic approach was evident, demonstrating a comprehensive PCC approach. Studies show that incorporating nurse‐led (telephone) follow‐up may not only improve patient outcomes but can also be cost‐effective compared to HFU [28, 29, 30, 31, 55, 56, 57]. We want to express our appreciation for the dedication and expertise demonstrated by nurses, giving them the recognition they rightfully deserve.

## 5 Conclusion

In conclusion, this study of gynaecological cancer survivors' experiences of follow‐up during the Covid‐19 pandemic highlights the need for personalised and patient‐centred follow‐up care that promotes patient empowerment, and how this can be provided by a designated contact person. The findings emphasise the importance of tailored support, involvement of family members, addressing information gaps, and overcoming barriers to self‐management. Finally, it provides directions for approaching and optimising follow‐up care, both in potential future periods of resource scarcity and in routine clinical practice.

The diversity of alternative follow‐up strategies to HFU offers the opportunity to develop care delivery models that can meet the unique needs of individual patients with a patient‐centred approach to care. Future studies should evaluate whether alternative approaches to follow‐up are safe, effective, patient‐centred and promote patient empowerment.

## Author Contributions

**Esther M. Vermaas:** conceptualisation, methodology, investigation, visualisation, writing – original draft. **Luc R. C. W. Van Lonkhuijzen:** conceptualisation, resources, supervision, writing – review & editing. **Henrike Westerveld:** resources, writing – review & editing. **Maaike A. Van der Aa:** conceptualisation, supervision, writing – review & editing. **Tijmen J. Bonestroo:** resources, writing – review & editing. **Brigitte F. M. Slangen:** conceptualisation, resources, writing – review & editing. **Johanna W. M. Aarts:** conceptualisation, methodology, investigation, resources, visualisation, supervision, writing – review & editing.

## Ethics Statement

Ethical approval was granted by the Ethics Committee of the Amsterdam University Medical Centre (#FWA00032965).

## Preprint

A preprint has previously been published. Reference: Esther M. Vermaas, Luc R. C. W. Van Lonkhuijzen, Henrike Westerveld, Maaike A. Van der Aa, Tijmen J. Bonestroo, Brigitte F. M. Slangen, Johanna W. M. Aarts, The Patient's Voice on What We Can Learn From the COVID‐19 Pandemic About Gynaecologic Oncology Follow‐up Care: a Qualitative Study. 2024: Authorea.

## Conflicts of Interest

The authors declare no conflicts of interest.

## Supporting information


**Supplement 1:** ISSM_COREQ_Checklist.

**Supplement 2:** *Topic list: Focus groups follow‐up during COVID‐19*.

## Data Availability

The data that support the findings of this study are available from the corresponding author upon reasonable request.

## Endnotes

1 In this article, the term ‘women’ is used to refer to women and people with female reproductive organs, including but not limited to those with ovaries, uteri and/or vulvae. While the majority of patients in this field identify as women, we acknowledge that gynaecologic oncology care must be inclusive of all individuals with relevant anatomy and medical needs.
