# Supplementary material for: Patient Voices: What Can We Learn From the Covid‐19 Pandemic About Follow‐Up Care in Gynaecologic Oncology?
Source: Health Expect. 2025 Sep 22;28(5):e70405. doi: 10.1111/hex.70405 (PMC12451077; doi:10.1111/hex.70405)
Supplement: Supplementary file 2 — Supplement 2: Topic list: Focus groups follow‐up during COVID‐19. [file HEX-28-e70405-s001.pdf]

Supplement 2. Topic list: Focus groups follow-up during COVID-19

Frequency of follow-up visits (1-5)	Less follow-up visits (hospital care)	Patient experience	Match individual needs (individualised)	What would you have wanted differently, was there something you missed or wanted more of
			Own say in frequency of visits (flexible)	
		Therapeutic relationship	Satisfaction with amount of contact with care giver	
	Receiving less care from care takers outside the hospital (GP, paramedic care like physiotherapy, mental support care) (holistic)	Effect of not receiving enough support	Physically	
			Mentally	
Information (2, 4)	Receiving information			
	Missed information			
	Asking questions			
Telemedicine (6-9)	Convenience	Time-saving	Travel time	In hospital waiting time
		Cost-saving	Travel costs	
		Access to care	waiting times to an appointment when requesting one	Flexible (i.e. combining an appointment with work)
	Avoidance of infectious disease	Anxiety		
	Technical difficulties	Devices/software		
		Care givers technical skills		
		Patients technical skills		
	Therapeutic relationship	Telephone vs in-person contact	Video vs in-person contact	
		Own say in form of consultations		
	Health concerns	Patient perceived need for exam or testing		

1. Zola P, Ciccone G, Piovano E, Fuso L, Di Cuonzo D, Castiglione A, et al. Effectiveness of Intensive Versus Minimalist Follow-Up Regimen on Survival in Patients With Endometrial Cancer (TOTEM Study): A Randomized, Pragmatic, Parallel Group, Multicenter Trial. J Clin Oncol. 2022;40(33):3817-+.
2. Kennedy F, Shearsmith L, Holmes M, Peacock R, Lindner OC, Megson M, et al. 'We do need to keep some human touch'-Patient and clinician experiences of ovarian cancer follow-up and the potential for an electronic patient-reported outcome pathway: A qualitative interview study. Eur J Cancer Care (Engl). 2022;31(2):e13557.
3. de Rooij BH, Ezendam NPM, Nicolaije KAH, Vos MC, Pijnenborg JMA, Boll D, et al. Effects of Survivorship Care Plans on patient reported outcomes in ovarian cancer during 2-year follow-up - The ROGY care trial. Gynecol Oncol. 2017;145(2):319-28.
4. Lewis RA, Neal RD, Hendry M, France B, Williams NH, Russell D, et al. Patients' and healthcare professionals' views of cancer follow-up: systematic review. Br J Gen Pract. 2009;59(564):e248-59.
5. Jeppesen MM, Jensen PT, Hansen DG, Christensen RD, Mogensen O. Patient-initiated follow up affects fear of recurrence and healthcare use: a randomised trial in early-stage endometrial cancer. BJOG. 2018;125(13):1705-14.
6. Kraus EJ, Nicosia B, Shalowitz DI. A qualitative study of patients' attitudes towards telemedicine for gynecologic cancer care. Gynecol Oncol. 2022;165(1):155-9.
7. Mojdehbakhsh RP, Hurtado ACM, Uppal S, Milakovich H, Spencer RJ. The long game: Telemedicine patient satisfaction metrics and methods of recurrence detection for gynecologic cancer patients throughout the initial year of the COVID-19 pandemic. Gynecol Oncol Rep. 2022;42.
8. Zimmerman BS, Seidman D, Berger N, Cascetta KP, Nezolosky M, Trlica K, et al. Patient Perception of Telehealth Services for Breast and Gynecologic Oncology Care during the COVID-19 Pandemic: A Single Center Survey-based Study. J Breast Cancer. 2020;23(5):542-52.
9. Kumarakulasingam P, McDermott H, Patel N, Boutler L, Tincello DG, Peel D, et al. Acceptability and utilisation of patient-initiated follow-up for endometrial cancer amongst women from diverse ethnic and social backgrounds: A mixed methods study. Eur J Cancer Care (Engl). 2019;28(2):e12997.
